# Selective Upregulation of Interleukin 1 Receptor Antagonist and Interleukin-8 in Fuchs’ Endothelial Corneal Dystrophy with Accompanying Cataract

**DOI:** 10.3390/jcm13102815

**Published:** 2024-05-10

**Authors:** Rafał Fiolka, Edward Wylęgała, Michał Toborek, Jowita Adamczyk-Zostawa, Zenon P. Czuba, Adam Wylęgała

**Affiliations:** 1 Department of Ophthalmology, Faculty of Medical Sciences in Zabrze, Medical University of Silesia in Katowice, 40-055 Katowice, Poland; ewylegala@sum.edu.pl (E.W.); jowitkaa@gmail.com (J.A.-Z.); 2 Doctoral School of the Medical University of Silesia in Katowice, 40-055 Katowice, Poland; 3 Department of Biochemistry and Molecular Biology, University of Miami School of Medicine, Miami, FL 33136, USA; mtoborek@med.miami.edu; 4 Department of Microbiology and Immunology, Faculty of Medical Science, Zabrze Medical University of Silesia in Katowice, 40-055 Katowice, Poland; zczuba@sum.edu.pl; 5 Health Promotion and Obesity Management, Pathophysiology Department, Medical University of Silesia in Katowice, 40-752 Katowice, Poland; adam.wylegala@gmail.com

**Keywords:** cataract, Fuchs’ endothelial corneal dystrophy, IL-1Ra, inflammatory cytokines, anti-inflammatory cytokines

## Abstract

(1) **Background**: Patients with Fuchs’ endothelial corneal dystrophy (FECD) may have coexisting cataracts and, therefore, may require a cataract surgery, which poses challenges due to potential endothelial cell damage. FECD is a degenerative eye disease of unclear etiology, with inflammatory cytokines maybe playing an important role in its development and progression. The present study aimed to investigate the cytokine profile in the aqueous humor of FECD eyes with cataract. (2) **Methods**: Fifty-two patients were included in the study, 26 with FECD + cataract and 26 with cataract as a control group. Samples of the aqueous humor were analyzed for pro- and anti-inflammatory cytokines using a Bio-Plex 200 system. (3) **Results**: Interleukin 1 receptor antagonist (IL-1Ra) and interleukin IL-8 levels were significantly higher in the aqueous humor of FECD + cataract patients compared to the control/cataract group. Moreover, the levels of anti-inflammatory IL-10 showed a strong trend to be higher in the FECD + cataract group compared to the control group. In contrast, there were no statistically significant differences in IL-1β, IL-6, IL-4, IL-10, IL-13, IL-17A, and tumor necrosis factor TNF-α between the groups. (4) **Conclusions**: Presented research contributes to a better understanding of FECD pathogenesis. Elevated levels of IL-1Ra and IL-8 may serve as a defense mechanism in people with FECD and coexisting cataract.

## 1. Introduction

Fuchs’ endothelial corneal dystrophy (FECD) affects the corneal endothelium, which plays an important role in maintaining hydration homeostasis and cornea clarity, resulting in edema and corneal guttae, which are typical excrescences on the Descemet’s membrane [1,2]. As the changes progress, they can lead to dermis swelling, subsequent intra- and subepithelial blister formation, and subepithelial fibrosis with superficial corneal vascularization. Eventually, FECD damage to the cornea can result in corneal blindness [3].

FECD is classified as a rare, progressive, and hereditary disease with a female predilection, and may have an early or late onset due to a complex interplay of multiple genes. The CTG18.1 trinucleotide repeat expansion in the transcription factor 4 gene (TCF4) is the most prevalent genetic risk factor for classic, late-onset FECD, typically manifesting after the age of 40 [4]. Additionally, non-genetic factors such as UV exposure and smoking have been linked to FECD by increasing oxidative stress in corneal endothelial cells [5,6,7,8,9]. Currently, Descemet membrane endothelial keratoplasty (DMEK) is a gold standard for the FECD treatment [10,11].

The precise triggering mechanism of FECD remains unknown, but recent immunological studies suggest a role for pro- and anti-inflammatory cytokines in its pathogenesis. Elevated levels of cytokines like IL-1, IL-6, IL-8, and others in the aqueous humor have been implicated in stimulating inflammatory processes, tissue remodeling, and endothelial cell apoptosis [12,13,14,15].

Patients with FECD may have coexisting cataracts and, therefore, may require a cataract surgery, which poses challenges due to potential endothelial cell damage [16,17]. Endothelial damage can result in increased corneal decompensation, painful corneal blisters, and the possible need for penetrating keratoplasty in a vulnerable eye such as the eye with FECD [18,19]. Therefore, predicting which patients may experience corneal decompensation after cataract surgery is of utmost importance in clinical care.

The profile of pro- and anti-inflammatory cytokines in FECD remains not fully understood. Therefore, the aim of our study was to evaluate the inflammatory and anti-inflammatory cytokine profile in the aqueous humor of patients diagnosed with FECD and/or cataracts for better understanding of FECD pathogenesis and development.

## 2. Materials and Methods

### 2.1. Ethical Permissions

The study was approved by the Bioethical Committee of the Medical University of Silesia (SUM) with the reference number PCN/CBN/0022/KB1/84/21 (15 June 2021). The study was performed in accordance with the standards of the Declaration of Helsinki. The patients were informed about the procedure and potential risks before they were asked to participate. Written informed consent to participate in the study and publish the research results were obtained from all patients before the study started. Patients’ data were protected by a statement of General Data Protection Regulation. All samples have been anonymized and numbered to ensure the avoidance of bias.

### 2.2. Initial Examination and Inclusion/Exclusion Criteria

**Figure 1:**
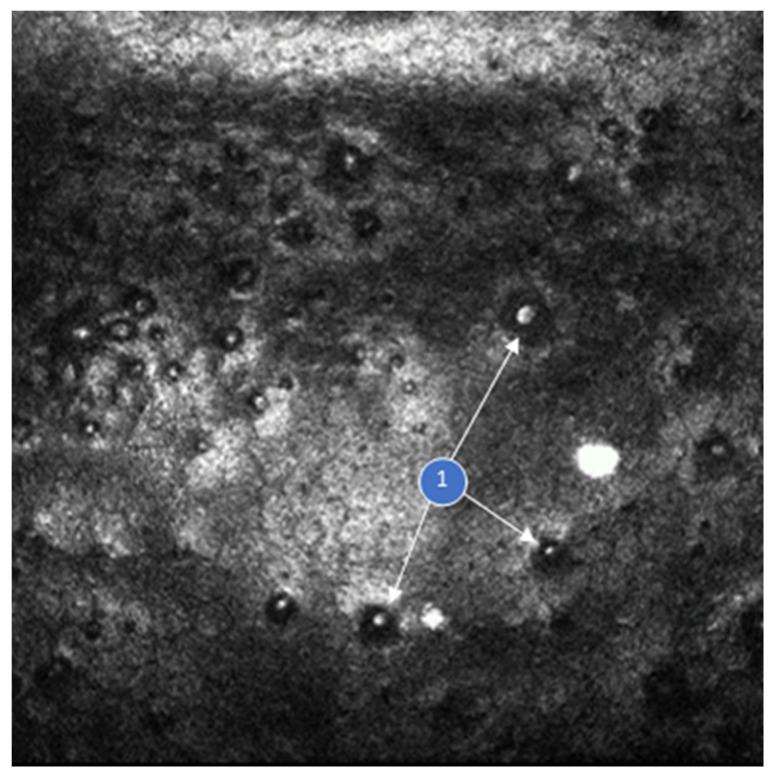
Corneal endothelium in Fuchs’ dystrophy [Department of Ophthalmology, Faculty of Medical Sciences in Zabrze, Medical University of Silesia, Katowice, Poland].

The diagnosis of FECD was based on a comprehensive exam and recognized criteria for diagnosis [20,21]. A complete ophthalmic history was obtained, and participants underwent a standard ophthalmic examination, including Optical Coherence Tomography (OCT) imaging assay using CASIA2 (Cornea and Anterior Segment OCT; TOMEY GmbH, Nuremberg, Germany), and SOCT REVO FC (SOCT, spectral optical coherent tomography; FC, Fundus Camera; OPTOPOL Technology Sp. z o.o., Zawiercie, Poland), and slit-lamp examination in vivo corneal confocal microscopy with photographic documentation (Figure 1). Inclusion criteria were diagnosis of FECD and/or cataract, and age >18 years old. Exclusion criteria included patients with previous or concomitant corneal disease, previous intraocular surgery, history or signs of glaucoma, pseudoexfoliation syndrome or glaucoma uveitis, inflammatory conditions of the uveal membrane of the eye, neovascular age-related macular degeneration or diabetes, steroid therapy, other eye injuries, pregnancy, or cancer.

In vivo corneal confocal microscopy (HRT3 RCM; Heidelberg Engineering GmbH Heidelberg, Germany) depicting Fuchs’ Endothelial Corneal Dystrophy (FECD) with a disrupted hexagonal cell pattern. The image reveals the noticeable loss of corneal endothelial cells and the presence of guttae (examples dark areas—number 1 and arrow).

### 2.3. Study Design

**Figure 2:**
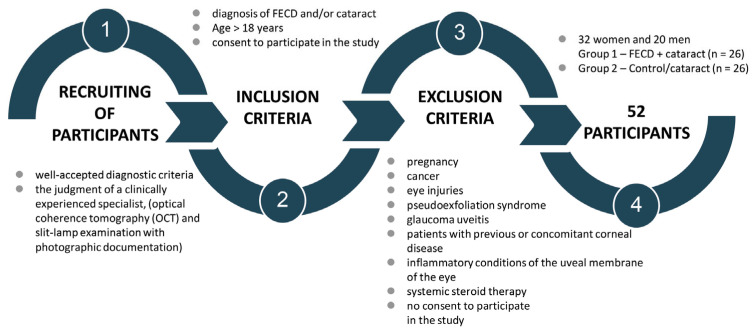
Study design—recruiting of participants, inclusion and exclusion criteria, study groups. Legend: OCT—optical coherence tomography; FECD—Fuchs’ endothelial corneal dystrophy.

The study was conducted from 2021 to 2023 in the Department of Ophthalmology, Faculty of Medical Sciences in Zabrze, Medical University of Silesia in Katowice, Poland. The study recruited 52 patients in total (Figure 2). Obtaining aqueous humor from healthy patients was not recommended by the Bioethics Committee; therefore, healthy eyes could not be included into the study as standard controls. The patients were divided into two groups:

1. The FECD + cataract group (n = 26). The group included patients with Fuchs’ endothelial corneal dystrophy and cataracts.
2. The control/cataract group (n = 26). The group included patients with cataracts only.

### 2.4. Sampled Material

To collect aqueous humor samples, eyelids and skin around the eye were wiped with disinfectant. Samples of 100 µL were aspirated using a 27-gauge ophthalmic needle through corneal paracentesis under general or local anesthesia at the beginning of cataract surgery. Samples were collected into sterile polypropylene tubes and were immediately frozen in −82 °C until further analyses. Collecting the aqueous humor did not adversely affect the cataract surgery and did not cause additional complications.

### 2.5. Biochemical Analyses

The biochemical analyses were carried out in blinded samples using the Bio-Plex 200 System from Bio-Rad and the Bio-Plex Pro Human Cytokine Panel 27-Plex (M500KCAF0Y). Representative assay performance characteristics were as follows: specificity, analyte cross-reactivity < 10%, intra-assay precision % CV (coefficient of variation) < 15%, accuracy, and percent recovery 70–130%. The Bio-Plex Suspension Array System included fluorescently labelled micro-spheres and instrumentation licensed to Bio-Rad Laboratories, Inc. by the Luminex Corporation (Austin, TX, USA). Standard curves were generated using the reference standards supplied with the kits and used to determine respective analyte concentrations for each sample. Minimum detection thresh-olds of individual analytes were IL-1β 0.1 pg/mL, IL-1Ra 4.0 pg/mL, IL-6 0.05 pg/mL, IL-8 0.05 pg/mL, and TNF-α 0.05 pg/mL, IL-4 0.01 pg/mL, IL-10 0.05 pg/mL, IL-13 0.05 pg/mL, and IL-17A 0.05 pg/mL.

### 2.6. Statistical Analysis

We employed Statistica v 13.3 (Tibco, San Francisco, CA, USA) software for statistical analysis. The Shapiro–Wilk test was used to assess normality, evaluating the distribution of each relevant variable. For comparative analysis, the non-parametric Mann–Whitney U test was applied to variables with non-parametric distribution, while the parametric *t*-test was used for parametric variables like age. The Chi-squared test was utilized to examine gender distribution differences between the groups. Statistical significance was assumed at a *p*-value < 0.05. A multiple regression test was used to evaluate the relationship between variables, with cytokine levels as the dependent variable and patient factors such as age, gender, IOP, and age as independent variables, in whole group of patients. The analysis was designed to verify the hypothesis that independent variables affect the concentration of cytokines, for which levels were found to be statistically significant differences between the study groups (FECD + cataract vs. control/cataract). The study sample size was calculated based on the means and standard deviations reported in the paper by Price et al. [22]. The accepted level of significance was set as *p* ≤ 0.05, with a wanted power of 90%. Using a sample size of 14 patients per group, the study would have had a power of 91.9% to yield statistically significant results.

## 3. Results

The cohort included 52 patients (32 females and 20 males) aged 71.77 ± 7.59 years undergoing cataract surgery. Distribution between females and males was non-significant (*p* = 0.09). There were no differences in age of patients between the groups (*p* > 0.05).

Table 1 represents important ophthalmic parameters in patients involved in the study groups with *p*-values between the groups.

The median visual acuity was 0.180 (range: 0.0200 to 1.000) and the median intraocular pressure was 15.00 mmHg (range: 11.00 to 20.00). The median spherical refractive error was 1.00 D (range: −14.00 to 7.00), while the median cylindrical refractive error was −0.75 D (range: −4.00 to 0.00) and the median axis was 70.0 (range: 0.00 to 179.00). The median corneal endothelial cell density was 2404.0 (range: 886.00 to 3331.00) and the median of the keratometry flat (K1) value was 43.89 (range: 29.51 to 59.80). The median axis of the K1 value was 75.0 (range: 0.00 to 179.00) and the median keratometry steep value (K2) was 44.54 D (range: 15.68 to 65.70). The axis value was 92.0 (range: 90.00 to 174.00). The median cylindrical power was −0.80 D (range: −18.1 to 0.00).

There was a statistically significantly lower measure of visual acuity (BCVA *p* < 0.001), cylinder resulting from the measurement of corneal curvature by keratometry (*p* < 0.001), and endothelial cell count (ECD, *p* < 0.01) in patients in the FECD + cataract group as compared to the control/cataract group. No statistically significant difference was observed between the groups in terms of IOP (*p* = 0.538). At the same time, a significant difference was found in the axis values between the two groups (*p* = 0.001). The FECD + cataract group had a median axis of 10.00 (Min. = 0.00, Max. = 170.00), whereas the control/cataract group had a median of 123.00 (Min. = 10.00, Max. = 179.00). No statistically significant difference was observed between the groups in terms of spherical equivalence (*p* = 0.138). Also, the groups showed no statistically significant difference in cylinder values (*p* = 0.194). No statistically significant difference was observed in keratometry 1 values between the two groups (*p* = 0.262). Similarly, no statistically significant difference was found in keratometry 1 axis values (*p* = 0.187). No statistically significant difference was observed in keratometry 2 and Keratometry axis values (*p* = 0.565) and (*p* = 0.889), respectively. A significant difference in cylinder values was noted between the groups (*p* = 0.001) as well as in axis values between the groups (*p* = 0.03).

**Figure 3:**
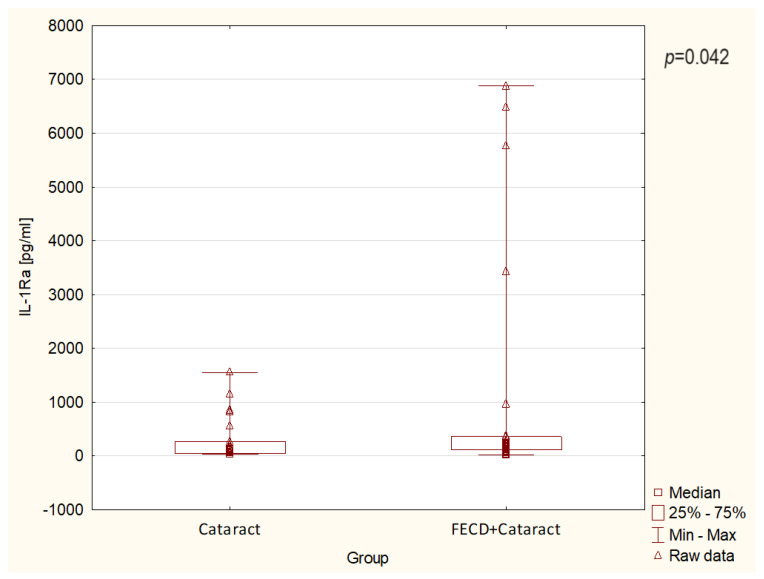
The levels of IL-1Ra in the aqueous humor in the study groups. The box represents the interquartile range (IQR), whiskers represent the minimum and maximum values, and triangles represent patients’ raw data. The differences between the groups are significant with *p* < 0.05.

**Figure 4:**
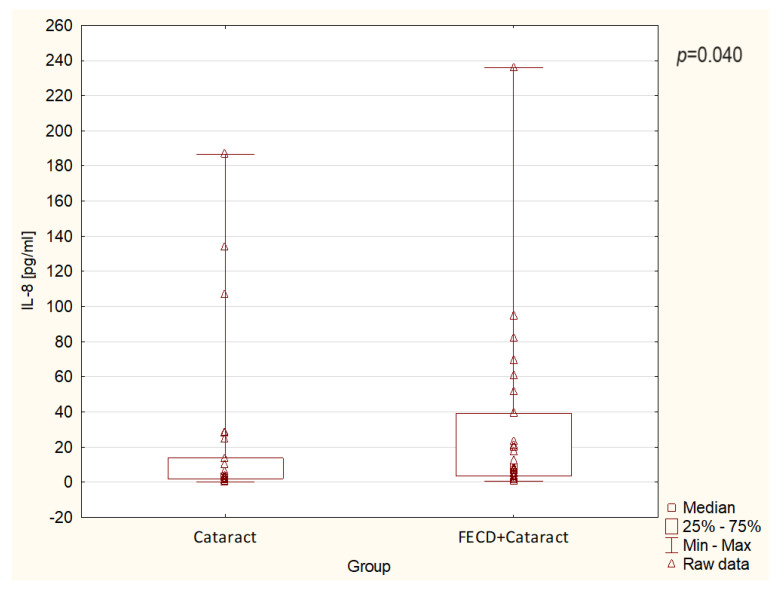
The levels of IL-8 in the aqueous humor in the study groups. The box represents the interquartile range (IQR), whiskers represent the minimum and maximum values, and triangles represent patients’ raw data. The differences between the groups are significant with *p* < 0.05.

The levels of inflammatory and anti-inflammatory cytokine in the FECD + cataract and the control/cataract groups are presented in detail in Figure 3 and Figure 4 and in Table 2. Among the parameters studied, IL-1Ra levels were significantly higher in the FECD + cataract group compared to the control/cataract group (*p* < 0.05) (Figure 3). Moreover, IL-8 levels were significantly higher in the FECD + cataract group compared to the control/cataract group (*p* < 0.05) (Figure 4). In contrast, inflammatory cytokines IL-1β, IL-6, IL-17A, and TNF-α, and anti-inflammatory cytokines: IL-4, IL-10, and IL-13 showed no statistically significant differences between the groups (Table 2), suggesting similarity in their levels between the FECD + cataract and control/cataract groups, with the exception of IL-10, where levels revealed a strong trend to be increased in the FECD + cataract study group compared to the control/cataract group.

The multiple regression results of IL-8 and IL-1Ra levels in the aqueous humor are presented in Table 3. Among the parameters studied, we observed a significant association between IOP and IL-8 concentration. There were no others significant associations observed.

## 4. Discussion

The transparent cornea of the eye, responsible for refraction, is the first mechanical barrier that protects the eye from the penetration of microorganisms, including pathogens. The lack of blood and lymphatics vessels, low number of macrophages, and the presence of the blood–ocular barrier make it immunologically privileged. The processes of tolerance and immunological ignorance prevent the onset of excessive inflammation, which could potentially cause irreversible damage to delicate eye structures and contribute to the development of severe inflammatory conditions and diseases [23,24,25,26,27,28].

Immunity of the immunologically privileged anterior chamber of the eye depends on the presence of aqueous humor (AH) with immunosuppressive and anti-inflammatory properties. It fills the anterior and posterior chambers of the eye and creates the intraocular pressure (IOP) [29]. In addition to chloride ions, lactate, and ascorbate, it also contains anti-inflammatory cytokines (IL-4, 7, 8, 10), proinflammatory cytokines (IL-1, 6, 12, TNF-α), chemokines (MCP-1, MIP-1α, MIP-1β, IFN-γ), and growth factors (G-CSF, GM-CSF) [30]. Changes in AH composition could be a reason for a pathological process developing in the anterior chamber of the eye. Chowdhury et al. showed that a change in concentration of endogenous biologically active substances in the AH is closely related to a decrease in corneal epithelial cell density that leads to endothelial dysfunction with prolonged stromal edema and bullous changes in the epithelium (bullous keratopathy) [29]. Moreover, Yagi-Yaguchi et al. showed that lower endothelial cell density is associated with elevated levels of IL-1α, IL-4, IL-13, MIP-1β, TNF-α, and E-selectin [13]. We previously identified higher levels of RANTES (CCL5), eotaxin CCL11), and IP-10 (CXCL10) in the aqueous humor of patients with FECD and cataract as compared to patients who suffered from cataract only [31]. These chemokines may also be associated with the progression of endothelial changes, leading to the development of FECD.

However, the profile of other pro- and anti-inflammatory cytokines in FECD remains not fully understood. So, we focused in the present study on evaluation of selected pro-inflammatory cytokines (IL-1β, IL-6, IL-8, IL-17A, and TNF-α), anti-inflammatory cytokines (IL-4, IL-10, and IL-13), and the IL-1 receptor antagonist (IL-1Ra) in the aqueous humor of FECD and/or cataract eyes. These factors regulate immune responses, while IL-8 is the main chemotactic factor. Together, they are involved in vital regulation of cellular processes and play an important role in the pathogenesis of a number of inflammatory diseases [27,32,33,34,35,36,37].

In our study, we did not observe any significant differences in anti-inflammatory interleukins levels between the study groups. Simultaneously, we detected significantly higher level of the IL-1Ra in the FECD + cataract group in comparison to the control/cataract group. This novel observation suggests a suppressive impact of IL-1Ra on IL-1β-mediated induction of inflammation, which may consequently lead to the development of cataracts in FECD eyes. Even more importantly, higher levels of IL-1Ra could be considered in the future, after more experiments are conducted, as an indicator to distinguish the FECD + cataract eyes from those with cataracts. Among studied inflammatory cytokines, we observed a significant increase only of IL-8 levels between the FECD + cataract group compared to the control/cataract group. We did not find any data suggesting that persistently higher levels of IL-8 in the AH of FECD patients can cause endothelial or corneal cell damage in FECD. Taking into account the obtained results, we suggest that higher levels of IL-8, a pro-inflammatory chemokine, may cause inflammatory cells to infiltrate the cornea and perhaps damage ocular structures [38,39]. In addition, a higher level of IL-8 in the eyes of FECD + cataract patients may also indicate its potential to predict the occurrence of FECD, but this observation needs further research.

There are only a few studies on the concentration of cytokines determined in the aqueous humor patients with FECD. Moreover, these reports are also conflicting. In addition, the literature studies were often conducted in eyes with corneal transplants, and not, as in our work, in patients without corneal transplants. This proves the need to conduct research in this direction, which could expand information on FECD pathogenesis and development.

Fishenko et al. evaluated cytokine levels in the aqueous humor of patients with FECD and bullous keratopathy (BK) compared to healthy, but not cataract, controls. Multiplex analysis indicated significantly higher levels of IL-6, IL-8, GM-CSF, IFN-γ, MCP-1, and MIP-1β in FECD eyes in comparison to healthy eyes. Similar to our finding, these authors also found no significant differences in the levels of pro-inflammatory cytokines IL-1β and TNF-α and a non-significant deviation of IL-4 and IL-13 levels in FECD [12]. The obtained data confirm that FECD is associated with disruption of ocular immune privilege that leads to chronic local inflammation, which, in turn, causes remodeling of the corneal tissues, resulting in fibrosis.

The literature data indicate also that the development of FECD is associated with decreasing endothelial cell density (ECD). It can be assumed that this factor may affect cytokine levels in the AH. Multivariate analyses conducted by Yagi-Yaguchi et al. showed that lower ECD was associated with elevated levels of IL-1α, IL-4, IL-13, MIP-1β, TNF-α, and E-selectin [13]. ECD was correlated with a history of laser iridotomy, the status of intraocular lens, and the number of previous surgeries, and elevated cytokine levels in the AH showed different correlations with these clinical factors. These results suggest that a change in the microenvironment in the anterior chamber causes long-term endothelial cell loss as a result of chronic inflammation with elevated levels of inflammatory cytokines. The authors collected aqueous samples from 157 patients who underwent corneal transplantation and cataract surgery. The study encompassed patients with FECD and those with other conditions including glaucoma and a history of previous intraocular surgeries [13].

In our study, we assembled a small cohort of patients who had not undergone ophthalmic surgery. While this poses a limitation, it also results in a more homogeneous group. We observed a decrease in ECD and higher visual acuity of the eye (BCVA) in the FECD + cataract study group compared to the cataract-only group. At the same time, we didn’t observe any difference in intraocular pressure between the groups. For the whole group of patients, we used multiple regression analysis, looking for associations between independent variables, such as ECD, IOP, age, and gender, and the determined cytokines. We did not observe associations between IL-8 and IL-1Ra and ECD, although endothelial cell density is directly related to the development of FECD. Regarding the other independent variables, only IOP appears to be significantly associated, but only with IL-8 concentration. Thus, it appears that an increase in IOP may be associated with an increase in corneal thickness and/or increased edema. We found no literature support for this observation. It certainly warrants further study. This lack of association may be attributed to the small size of our study group, limited to FECD patients with cataracts, and the homogeneity of the group, due to the exclusion criteria used. Drawing definitive conclusions at this stage is challenging. Nonetheless, we consider our findings preliminary, setting a foundation for future research.

Study limitations: We did not analyze other systemic variables in the study, such as systemic inflammation, or other health factors that could potentially affect cytokine concentrations. The eyes were characterized by immune privilege. Although the possibility of cytokines crossing the blood–eye barrier cannot be excluded, immune sequestration limits the movement of systemic cytokines into the eye, and especially into the AH. Moreover, generally, cytokines act over short distances in intercellular communication [40,41]. Therefore, systemic inflammation has a limited effect on cytokine levels in the eye, underscoring the diagnostic importance of inflammatory cytokines in the AH, as examined in our manuscript. The obtained results are innovative but require further research on a larger group of patients. The relatively small size of the study groups employed in the present pilot study resulted from the fact that FECD is a relatively rare ocular disease. Therefore, a multisite clinical research study is recommended for establishing reliable biomarkers of FECD.

## 5. Conclusions

The presented research contributes to a better understanding of FECD pathogenesis. Elevated levels of IL-1Ra and IL-8 may serve as a defense mechanism in people with FECD and coexisting cataract. Given the results of the multiple regression analysis, further studies should pay special attention to the association between FECD and cataracts with intraocular pressure (IOP).

## Figures and Tables

**Table 1:** Ophthalmic parameters measured in patients in the study groups with *p*-values between the groups.

	FECD + Cataract (n = 26)					Cataract/Control (n = 26)					
Variable	Median	Min.	Max.	Lower Quartile	Upper Quartile	Median	Min.	Max.	Lower Quartile	Upper Quartile	*p* Value
BCVA	0.10	0.02	0.60	0.06	0.16	0.40	0.02	1.00	0.2	0.6	0.001
IOP	15.00	110.00	20.00	14.00	17.00	15.00	12.00	18.00	14.00	16.00	0.538
ECD	1282.0	886.00	1395.00	886.00	1395.00	2452.0	1908.00	3331.00	2186.00	2657.00	0.01
Age	72.3	59.00	87.00	67.00	79.00	71.19	55.00	89.00	67.00	74.00	0.59

Legend: BCVA—measure of visual acuity (Best Corrected Visual Acuity); IOP—intraocular pressure [mmHg]; ECD—endothelial cells count [cells/mm^2^]; Age—in [years].

**Table 2:** Cytokine levels in the aqueous humor among the studied groups.

	FECD + Cataract Group (n = 26)					Control/Cataract Group (n = 26)					
Cytokine [pg/mL]	Median	Min.	Max.	Lower Quartile	Upper Quartile	Median	Min.	Max.	Lower Quartile	Upper Quartile	*p* Value
IL-1Ra	214.45	25.12	6879.99	118.03	359.62	108.17	32.62	1551.50	40.82	266.48	0.04
IL-1β	1.49	0.90	3.15	1.35	1.49	1.49	0.15	3.42	1.22	2.70	0.35
IL-6	11.88	1.79	822.53	4.76	177.61	12.59	0.09	656.70	1.42	185.14	0.37
IL-8	8.01	0.61	236.01	3.80	39.38	3.05	0.06	186.76	2.08	13.67	0.04
TNF-α	0.59	0.09	8.16	0.53	1.73	0.62	0.14	6.48	0.53	1.06	0.70
IL-4	0.06	0.03	0.77	0.06	0.27	0.07	0.01	0.69	0.05	0.11	0.75
IL-10	0.86	0.19	2.82	0.28	2.01	0.56	0.10	2.14	0.28	0.86	0.12
IL-13	0.15	0.09	1.96	0.13	0.81	0.14	0.02	1.62	0.13	0.26	0.59
IL-17A	0.68	0.31	2.43	0.46	0.91	0.68	0.08	1.36	0.46	0.91	1.00

Legend: IL-1Ra—Interleukin 1 antagonist; IL-1β—interleukin 1 beta; IL-6—interleukin 6; IL-8—interleukin 8; TNFα—Tumor necrosis factor alpha, IL-4—interleukin 4, IL-10—interleukin 10, IL-13—interleukin 13, IL-17A—interleukin 17A.

**Table 3:** Multiple regression results of cytokine levels in the aqueous humor.

Dependent Variable Concentration	Independent Variable	Unstandardized Coefficients	SE	*p*-Value
IL-1Ra	IOP	51,446,651.267	86,676,520.435	0.584
	ECD	−60,273.732	255,221.358	0.329
	Age	−1,408,101.588	17,329,352.794	0.838
	Gender	−286,714,585.112	259,605,453.119	0.196
	Group	−721,819,517.573	656,708,840.517	0.283
IL-8	IOP	125,092,339.633	55,694,320.373	0.034
	ECD	−7245.852	163,993.432	0.965
	Age	−5,260,888.285	11,135,040.049	0.641
	Gender	−111,965,039.184	166,810,448.827	0.508
	Group	−229,175,845.519	430,265,817.348	0.599

Legend: SE—standard error; IOP—intraocular pressure [mmHg]; ECD—endothelial cells count [cells/mm^2^]; Age—in [years], Group—whole group of patients (FECD + cataract and control/cataract); IL-1Ra—Interleukin 1 antagonist; IL-8—interleukin 8.

## Data Availability

The database of aggregated statistics ready for analysis is stored in a secure and password-protected repository on the server of the Medical University of Silesia. The data were anonymized. Completely non-identifiable records are available to interested persons/organizations upon request from the corresponding author at fiolkarafal@gmail.com.
